# Novel Genotypes of Nidicolous *Argas* Ticks and Their Associated Microorganisms From Spain

**DOI:** 10.3389/fvets.2021.637837

**Published:** 2021-03-29

**Authors:** Ana M. Palomar, Jesús Veiga, Aránzazu Portillo, Sonia Santibáñez, Radovan Václav, Paula Santibáñez, José A. Oteo, Francisco Valera

**Affiliations:** ^1^Centre of Rickettsiosis and Arthropod-Borne Diseases, Hospital Universitario San Pedro-Center for Biomedical Research of La Rioja (CIBIR), Logroño, Spain; ^2^Departamento de Ecología Funcional y Evolutiva, Estación Experimental de Zonas Áridas -Consejo Superior de Investigaciones Científicas (EEZA-CSIC), Ctra. de Sacramento s/n, La Cañada de San Urbano, Almería, Spain; ^3^Institute of Zoology, Slovak Academy of Sciences, Bratislava, Slovakia

**Keywords:** soft ticks, *Argas* spp., nidicolous, cavity-nesting birds, tick-borne bacteria, tick-borne viruses, tick-borne protozoa, Spain

## Abstract

The knowledge of the distribution, richness and epidemiological importance of soft ticks of the genus *Argas* is incomplete. In Spain, five *Argas* species have been recorded, including three ornitophilic nidicolous ticks, but their associated microorganisms remain unknown. This study aimed to investigate ticks from bird nests and their microorganisms. Ticks were collected extensively from natural cavities and nest-boxes used by European rollers (*Coracias garrulus*) and little owls (*Athene noctua*) in Southeastern and Central Spain. Ticks were morphologically and genetically identified and corresponding DNA/RNA tick extracts were analyzed [individually (*n* = 150) or pooled (*n* = 43)] using specific PCR assays for bacteria (Anaplasmataceae, *Bartonella, Borrelia, Coxiella*/*Rickettsiella*, and *Rickettsia* spp.), viruses (Flaviviruses, Orthonairoviruses, and Phenuiviruses), and protozoa (*Babesia*/*Theileria* spp.). Six *Argas* genotypes were identified, of which only those of *Argas reflexus* (*n* = 8) were identified to the species level. *Two* other genotypes were closely related to each other and to *Argas vulgaris* (*n* = 83) and *Argas polonicus* (*n* = 33), respectively. These two species have not been previously reported from Western Europe. Two additional genotypes (*n* = 4) clustered with *Argas persicus*, previously reported in Spain. The remaining genotype (*n* = 22) showed low sequence identity with any *Argas* species, being most similar to the African *Argas africolumbae*. The microbiological screening revealed infection with a rickettsial strain belonging to *Rickettsia fournieri* and *Candidatus* Rickettsia vini group in 74.7% of ticks, mainly comprising ticks genetically related to *A. vulgaris* and *A. polonicus*. Other tick endosymbionts belonging to *Coxiella, Francisella* and *Rickettsiella* species were detected in ten, one and one tick pools, respectively. In addition, one *Babesia* genotype, closely related to avian *Babesia* species, was found in one tick pool. Lastly, Anaplasmataceae, *Bartonella, Borrelia*, and viruses were not detected. In conclusion, five novel *Argas* genotypes and their associated microorganisms with unproven pathogenicity are reported for Spain. The re-use of nests between and within years by different bird species appears to be ideal for the transmission of tick-borne microorganisms in cavity-nesting birds of semiarid areas. Further work should be performed to clarify the taxonomy and the potential role of soft *Argas* ticks and their microorganisms in the epidemiology of zoonoses.

## Introduction

Soft ticks of the genus *Argas* Latreille, 1795 (Ixodida; Argasidae) are distributed worldwide and include around 60 species (1). Of them, only eight species have been described in the Western Palearctic region, specifically, *Argas gilcolladoi, Argas persicus, Argas reflexus, Argas transgariepinus, Argas vespertilionis, Argas macrostigmatus, Argas vulgaris*, and *Argas polonicus* (2–5). All but the latter three species have been reported in Spain (Southwestern Europe) as parasites of birds or bats (6). The majority of *Argas* spp. are nidicolous and birds are exclusive vertebrate hosts for several species, mainly those of *Persicargas* subgenera, while humans are accidental hosts (7, 8). The genus *Argas* includes species responsible for the transmission of pathogens of medical and veterinary interest. Apart from conditions caused directly by soft ticks, such as toxicosis and anaphylaxis (9, 10), these ticks carry microorganisms that could be agents of infectious diseases. Specifically, *Argas* species can vector bacterial pathogens such as *Borrelia anserina* and *Aegyptianella* spp. and viruses such as Issyk-kul virus (11, 12). Other microorganisms with unproved pathogenicity have been detected in *Argas* ticks: bacteria from genera *Anaplasma, Bartonella, Borrelia, Coxiella, Ehrlichia, Francisella, Rickettsia*, and *Rickettsiella*, viruses belonging to Flaviviridae, Orthomyxoviridae, Orthonairoviridae, Phenuiviridae, and Reoviridae families, and protozoans such as *Babesia* and *Hemolivia* spp. (7, 13–17).

The lack of information on the natural history and distribution of various *Argas* species, their incorrect or incomplete taxonomic description, and the fact that some species share morphological features but have not been molecularly examined, are responsible for the poor knowledge of *Argas* ticks in Spain. Moreover, their role in the epidemiology of tick-borne microorganisms has not been studied in this country. Here, we aim at describing soft ticks from natural and artificial nests occupied by different cavity-nesting birds in Spain and the prevalence of selected tick-borne microorganisms.

## Materials and Methods

### Study Area and Study System

The main study area (~50 km^2^) lies in the Desert of Tabernas (Almería province, SE Spain, 37.08°N, 2.35°W). The landscape mostly consists of open shrubland with olive and almond groves interspersed among numerous dry riverbeds—ramblas. The climate in this area is semiarid Mediterranean with a marked water deficit during long, hot summer months. The mean annual rainfall is ~ 230 mm, with high inter- and intra-annual variability (18). Tick samples also were collected in Segovia and Guadalajara provinces (both in the interior of the Iberian Peninsula), whose climate is Mediterranean with some continental characteristics.

In the main study area in Almería, natural cavities in sandstone cliffs, seminatural cavities in stone bridges and abandoned farmhouses and nest boxes provide nest sites for cavity-nesting birds, namely the European roller (*Coracias garrulus*, hereafter roller), the little owl (*Athene noctua*) and the rock/feral pigeon (*Columba livia*, hereafter pigeon). In this study, we sampled ticks in cavities occupied by rollers and little owls. The roller is a migratory bird that arrives at its breeding grounds in the study area during the second fortnight of April whereas the little owl is a resident bird. Both species rear a single brood per year (19). In contrast to these species, the pigeon is a resident bird that breeds at any time of the year in our study area and does not use nest boxes. Other species breeding in natural and seminatural cavities mainly include jackdaws (*Corvus monedula*), and common kestrels (*Falco tinnunculus*), whereas Scops owls (*Otus scops*), spotless starlings (*Sturnus unicolor*), and house sparrows (*Passer domesticus*) can breed in nest boxes.

Given nest-site limitation in the study area, both intra- and interspecific competition for suitable nesting holes occur and individual cavities can be re-used by different species both within and between years. This is frequently the case in Almería, so that many samples were collected from natural and seminatural cavities of rollers and little owls previously used by pigeons. The samples from Segovia were collected from a natural tree hole occupied by rollers but excavated by the Iberian green woodpecker (*Picus sharpei*), whereas the samples from Guadalajara were taken from rollers breeding in nest boxes.

### Tick Collection and Preservation

In the framework of a long-term project of cavity-nesting birds in the Desert of Tabernas, cavities and nest boxes have been routinely inspected during each breeding season since 2005 and both nestlings and nest material periodically examined for ectoparasites. Ticks were collected from cavities occupied by breeding rollers and little owls during 2009, 2012, 2015, and 2018–2020. Additionally, four tick individuals were obtained from roller nests in Central Spain (Guadalajara and Segovia) in 2004 (Table 1). Ticks collected until 2018 were preserved in ethanol, while ticks obtained in 2019 and 2020 were kept fresh upon delivery to the Centre of Rickettsiosis and Arthropod-borne Diseases (CRETAV). Before frozen at −80°C until later analysis, fresh ticks were identified and a single leg of each specimen was dissected.

**Table 1:** Tick samples used in this study.

**Province**	**Municipal boundary**	**Nest** **Coordinates**	**Date of collection**	**Host**	**Preservation method**	**Developmental stage/Gender**	**No. of specimens (No. of pools)**	**Tick species**
Almería	Tabernas	Diego tronco 37°3'58.43“N;2°21'19.38”W	17/06/2018	*Coracias garrulus*	Ethanol	Larvae	2 (1)	*Argas* sp. EEZA-CRETAV3
		Redondo Paloma 37° 3'58.39“N;2°21'19.48”W	27/06/2020	*C. garrulus*	Fresh-Frozen	Larvae	7 (1)	*Argas* sp. EEZA-CRETAV3
			28/06/2020	*C. garrulus*	Fresh-Frozen	Nymph	1 (1)	*Argas reflexus*
					Fresh-Frozen	Larvae	10 (2)	*Argas* sp. EEZA-CRETAV3
		RG 2M 37°4'14.86“N;2°20'27.65”W	17/06/2009	*C. garrulus*	Ethanol	Nymphs	2 (1)	*Argas* sp. EEZA-CRETAV3
		RH Grieta 37°3'53.50“N,2°20'48.89”W	2012	*C. garrulus*	Ethanol	Male	1 (1)	*Argas* sp. EEZA-CRETAV2
		RH SV 37°3'55.46“N;2°20'34.25”W	31/05/2012	*C. garrulus*	Ethanol	Nymph	1 (1)	*Argas* sp. EEZA-CRETAV1
		Tapadera alberca 37°3'54.53“N;2°21'30.43”W	01/06/2018	*Athene noctua*	Ethanol	Nymphs	7 (2)	*A. reflexus*
		Tapadera cueva 37° 3'56.71“N;2°21'24.29”W	08/06/2015	*C. garrulus*	Ethanol	Nymph	1 (1)	*Argas* sp. EEZA-CRETAV3
			09/05/2018	*A. noctua*	Ethanol	Adults or nymphs (last stage)	3 (1)	*Argas* sp. EEZA-CRETAV1
						Nymphs	6 (2)	*Argas* sp. EEZA-CRETAV1
						Nymphs	3 (1)	*Argas* sp. EEZA-CRETAV2
			08/06/2018	*A. noctua*	Ethanol	Adult	1 (1)	*Argas* sp. EEZA-CRETAV1
						Nymphs	3 (1)	*Argas* sp. EEZA-CRETAV1
			23/05/2019	*A. noctua*	Fresh-Frozen	Nymphs	12 (2)	*Argas* sp. EEZA-CRETAV1
						Nymphs	4 (1)	*Argas* sp. EEZA-CRETAV2
			30/05/2019	*A. noctua*	Fresh-Frozen	Nymph	1 (1)^a^	Argas sp. EEZA-CRETAV1
						Nymphs	2 (1)^b^	*Argas* sp. EEZA-CRETAV2
			10/06/2019	*A. noctua*	Fresh-Frozen	Male	1 (1)^a^	*Argas* sp. EEZA-CRETAV1
						Nymph	1 (1)^b^	*Argas* sp. EEZA-CRETAV2
			17/06/2019	*A. noctua*	Fresh-Frozen	Larva	1 (1)^a^	*Argas* sp. EEZA-CRETAV1
						Nymphs	2 (1)^a^	*Argas* sp. EEZA-CRETAV1
			13/05/2020	*A. noctua*	Fresh-Frozen	Nymphs	5 (1)	*Argas* sp. EEZA-CRETAV1
						Male	1	*Argas* sp.^*^
						Females	2	*Argas* sp.^*^
			27/05/2020	*A. noctua*	Fresh-Frozen	Nymphs	7 (1)	*Argas* sp. EEZA-CRETAV1
						Nymph	1 (1)^c^	*Argas* sp. EEZA-CRETAV2
			11/06/2020	*A. noctua*	Fresh-Frozen	Adult	1 (1)^c^	*Argas* sp. EEZA-CRETAV2
						Nymphs	5 (1)^c^	*Argas* sp. EEZA-CRETAV2
						Adult	1 (1)^d^	*Argas* sp. EEZA-CRETAV1
						Nymphs	12 (2)^d^	*Argas* sp. EEZA-CRETAV1
			15/06/2020	*A. noctua*	Fresh-Frozen	Larva	1	*Argas* sp.^*^
						Female	1	*Argas* sp.^*^
						Nymphs	12 (2)	*Argas* sp. EEZA-CRETAV2
						Nymphs	25 (5)	*Argas* sp. EEZA-CRETAV1
						Nymphs	8	*Argas* sp.^*^
	Tahal	Tahal cantera 37°8'7.13“N;2°14'22.39”W	18/06/2018	*C. garrulus*	Ethanol	Nymphs	1 (1)	*Argas* sp. EEZA-CRETAV2
	Uleila del Campo	Aguador NB cjo 37° 9'52.69“N;2°12'13.49”W	14/06/2015	*C. garrulus*	Ethanol	Males	2 (2)	*Argas* sp. EEZA-CRETAV1
						Nymph	1 (1)	*Argas* sp. EEZA-CRETAV2
						Female	1 (1)	*Argas* sp. EEZA-CRETAV2
Guadalajara	Illana	Chopera Illana 40°12'20.37“N;2°58'39.92”W	05/07/2004	*C. garrulus*	Ethanol	Males	2 (2)	*Argas* sp. EEZA-CRETAV5
Segovia	Pinarejos	Pinarejos 41°14'53.78“N;4°18'31.67”W	15/07/2004	*C. garrulus*	Ethanol	larvae	2 (2)	*Argas* sp. EEZA-CRETAV4

* not processed;

a,b,c,d: *same pool*.

*Data about sample origin (province, municipality, coordinates of the nest where individuals were captured), collection date, avian host, method of tick preservation, developmental stage, and gender (when possible), No. of ticks and No. of pools formed, and tick taxon name are given*.

### Tick Identification

The taxonomic identification of the ticks was carried out using morphological keys (20–23). Tick individuals were surface-sterilized and DNA was individually extracted from a single leg of each tick specimen using incubations with ammonium hydroxide (24). The obtained DNA templates were used for genetic characterization by the amplification and sequencing of the 16S rRNA fragment gene (25). Two other mitochondrial genes, 12S rRNA, and cytochrome oxidase subunit I (COI), were also used in analyses (Supplementary Table 1).

### Microbial Screening

Ticks were pooled (from 1 to 7 specimens; whole larvae and body halves for the other life stages) according to tick species or genotype, origin and date of collection and, when possible, tick developmental stage. DNA extracts from pools of ticks preserved in ethanol were obtained using the Qiagen DNA DNeasy blood and tissue kit (Qiagen, Hilden, Germany), following the manufacturer's recommendations. Ticks of each pool that were preserved frozen were homogenized in 600 μL of culture medium with antibiotics [Dulbecco's Modified Eagle Medium (DMEM) with 100 units/mL penicillin and 100 μg/mL streptomycin, Sigma]. Four hundred μL of the homogenate were used for nucleic acid extraction (DNA and RNA) and the remaining 200 μL were preserved at −80°C for future analysis. The DNA and RNA were extracted using the DNeasy blood and tissue kit and RNeasy Mini kit (Qiagen, Hilden, Germany), respectively, following the manufacturer's recommendations.

The quality of nucleic acid extraction was checked using the 16S rRNA PCR assay (25). Positive samples were subjected to microbial screening using specific PCR assays for the analysis of (i) bacteria: Anaplasmataceae family, *Bartonella* spp., *Borrelia* spp., *Coxiella* spp., *Rickettsiella* spp., and spotted fever group (SFG) *Rickettsia* spp., (ii) viruses: Flaviviridae, Orthonairoviridae and Phenuiviridae families, and (iii) protozoans: *Babesia* and *Theileria* spp. Negative and positive controls (DNA or cDNA extracts of *Anaplasma phagocytophilum, Bartonella henselae, Borrelia spielmanii, Borrelia miyamotoi, Coxiella*-like of *Rhipicephalus bursa, Rickettsia amblyommatis*, Crimean-Congo haemorrhagic fever virus, Japanese encephalitis virus, Uukuniemi uukuvirus virus, and *Babesia* sp. from *Rhipicephalus microplus*) were included in all the PCR assays performed. Primers and PCR conditions are described in the Supplementary Table 1. The SFG *Rickettsia* was screened in tick legs for all ticks. In addition, pools formed by specimens that gave negative results were also tested for *Rickettsia* spp. Moreover, all the pools were screened for the presence of the remaining bacteria and protozoans. Lastly, the viral screening was performed on tick pools comprising specimens of fresh/frozen ticks (Table 1).

### Prevalence of Infection

The prevalence of infection (PI) was estimated by:

PI = (No. of positive ticks/total No. of ticks analyzed) × 100%.

When microorganisms were amplified from pools of more than one tick, prevalence was calculated assuming that each positive pool contained only one positive tick. This estimate, known as minimum infectious rate (MIR), is calculated as follow:

MIR = (No. of positive pools/total No. of individual ticks analyzed) × 100%.

### Analysis of Nucleotide Sequences

Nucleotide sequences were analyzed using the BLAST search (https://blast.ncbi.nlm.nih.gov/Blast.cgi), and the resulting sequences were submitted to GeneBank. The Clustal Omega online software (https://www.ebi.ac.uk/Tools/msa/clustalo/) was used for multiple sequence alignment. Phylogenetic analyses were conducted with MEGA X (http://www.megasoftware.net) using the maximum likelihood method including all sites. The nucleotide substitution model was selected according to the Akaike information criterion implemented in MEGAX. Confidence values for individual branches of the resulting trees were determined by bootstrap analysis with 500 replicates.

## Results

### Tick Identification

**Figure 1:**
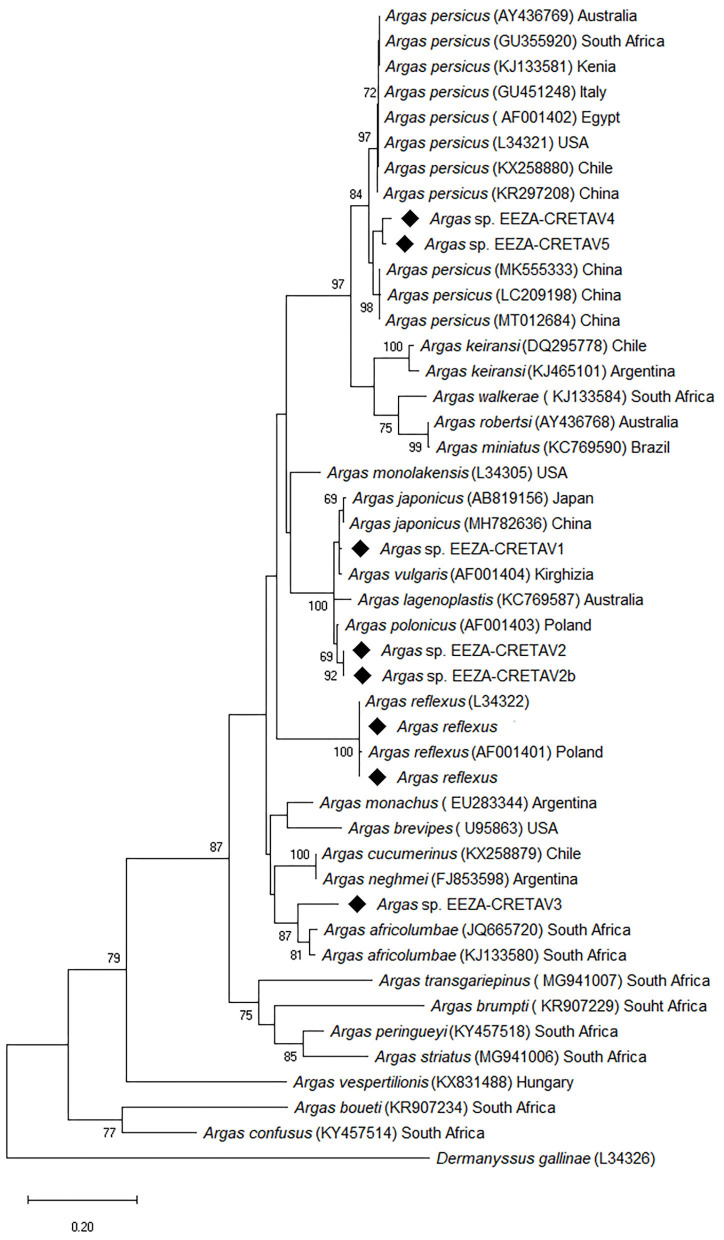
Phylogenetic tree based on 16S rRNA analysis showing the relationships between tick species and genotypes identified in this study and published validated *Argas* species. The evolutionary analysis was inferred using the maximum likelihood method and general time reversible + G model with Mega X. The analysis involved 46 nucleotide sequences and a total of 441 positions in the final dataset. The tree is drawn to scale, with branch lengths measured in the number of substitutions per site. Numbers (>65%) shown at the nodes correspond to bootstrapped percentages (for 500 repetitions). The GenBank accession number of sequences used in the analysis is shown in brackets after *Argas* taxon name and before sample origin. Sequences obtained in this study are marked with diamond. *Dermanysus gallinae* is used as outgroup.

One hundred and sixty-three ticks, mainly nymphs, were collected from bird nests in Almería (*n* = 159), Guadalajara (*n* = 2), and Segovia (*n* = 2). Arthropods were obtained from nest material in cavities occupied by little owl (*n* = 129) and roller (*n* = 34) or, in few cases, from nestlings of these species (Table 1). All the specimens were morphologically identified as *Argas* spp. and 150 specimens were further studied molecularly. Examination of morphological characters enabled the identification of eight nymphs as *A. reflexus*, but the morphological identification of the remaining ticks could not be accurately performed with available keys. The *A. reflexus* identification was corroborated molecularly based on 16S rRNA fragment gene (Table 2). The molecular identification was not conclusive for the remaining 142 tick samples, which were grouped based on the 16S rRNA results into five different genotypes, designated as *Argas* spp. EEZA-CRETAV1–5 (Tables 1, 2). Based on 16S rRNA gene analyses, *Argas* sp. EEZA-CRETAV1 (*n* = 83) and *Argas* sp. EEZA-CRETAV2 (*n* = 33) were closest to *A. vulgaris* and *A. polonicus*, respectively (Table 2). In turn, the *Argas* sp. EEZA-CRETAV3 genotype (*n* = 22) shared the highest identity (<92.2%) with *Argas africolumbae*. Lastly, *Argas* sp. EEZA-CRETAV4 (*n* = 2) and *Argas* sp. EEZA-CRETAV5 genotypes (*n* = 2) shared highest identities with *A. persicus* (Table 2). The phylogeny inferred from 16S rRNA analysis corroborates the BLAST results (Figure 1). Phylogenetic analyses based on 12S rRNA and COI fragment genes could not be performed because of the lack of homologue sequences for the majority of *Argas* spp.

**Table 2:** Highest similarities of the *Argas* genotypes detected in this study reached with public sequences from GenBank.

	**Fragment gene; GenBank accession No**.	**Identity (%)**	**Tick species (GenBank accession No.)**
*Argas reflexus*	16S rRNA; MW289075^a^	100	*A. reflexus* (L34322)
	12S rRNA; MW289084	96.6	*A. reflexus* (U95865)
	COI; MW288388^b^	81.5	*A. walkerae* (KJ133584)^h^
*Argas* sp. EEZA-CRETAV1	16S rRNA; MW289069	90.0	*A. vulgaris* (AF001404)
	12S rRNA; MW289077	94.1	*A. lagenoplastis* (KC769587)^i^
	COI; MW288380^c^	88.0	*A. lagenoplastis* (KC769587)^i^
*Argas* sp. EEZA-CRETAV2	16S rRNA; MW289070^d^	98.8	*A. polonicus* (AF001403)
	12S rRNA; MW289078^e^	93.8	*A. lagenoplastis* (KC769587)^i^
	COI; MW288382^f^	88.3	*A. lagenoplastis* (KC769587)^i^
*Argas* sp. EEZA-CRETAV3	16S rRNA; MW289072	92.1	*A. africolumbae* (JQ665720)
	12S rRNA; MW289081^g^	92.1	*A. africolumbae* (KJ133580)
	COI; MW288385	85.3	*A. africolumbae* (KJ133580)
*Argas* sp. EEZA-CRETAV4	16S rRNA; MW289073	96.5	*A. persicus* (MT012684)
	12S rRNA; MW289083	95.3	*A. persicus* (MT012684)
	COI; MW288386	90.1	*A. persicus* (KJ133581)
*Argas* sp. EEZA-CRETAV5	16S rRNA; MW289074	97.3	*A. persicus* (MT012684)
	12S rRNA; Not obtained		
	COI; MW288387	90.7	*A. persicus* (MT012684)

a *One more sequence with a single nucleotide substitution was obtained (MW289076)*.

b *One more sequence with two nucleotide substitutions was obtained (MW288389)*.

c *One more sequence with four nucleotide substitutions was obtained (MW288381)*.

d *One more sequence with a single nucleotide substitution was obtained (MW289071)*.

e *Two more sequences with one and three nucleotide substitutions were obtained (MW289079; MW289080)*.

f *Two more sequences with 20 and 16 nucleotide substitutions were obtained (MW288383; MW288384)*.

g *One more sequence with two nucleotide substitutions was obtained (MW289082)*.

h *There are not public sequences for A. reflexus*.

i *There are not public sequences for A. vulgaris and A. polonicus*.

*Argas* spp. EEZA-CRETAV1–3 specimens were collected in Almería, whereas those of *Argas* spp. EEZA-CRETAV4–5 were obtained in Segovia and Guadalajara. Also, some of the ticks of *Argas* spp. EEZA-CRETAV1–2 genotypes were collected from the same nests (Table 1).

### Bacterial Screening

All the DNA extracts (individual samples and pools) gave positive results for the tick-16S rRNA PCR assay and, consequently, were screened for bacteria (Table 3). Amplicons for *ompA* gene were obtained from 112 DNA extracts from tick legs (PI = 74.7%). Specifically, *Rickettsia* was amplified from 81 and 31 samples belonging to *Argas* sp. EEZA-CRETAV1 (PI = 97.6%) and *Argas* sp. EEZA-CRETAV2 (PI = 93.9%) samples, respectively. DNA extracts corresponding to individual tick-leg samples from *A. reflexus* and *Argas* sp. EEZA-CRETAV3–5 specimens were negative, but body-halve extracts of these specimens were further analyzed using pooled samples. The nucleotide extracts from these pools were negative for all *A. reflexus* specimens, but yielded positive results for two out of six pools of *Argas* sp. EEZA-CRETAV3 specimens (MIR = 9.1%) (comprising a nymph collected from a roller nest in Almería in 2015 and larvae that were attached to a roller nestling in Almería in 2020), and all the pools of *Argas* sp. EEZA-CRETAV4–5 specimens (MIR = 100% for the two pools) (Table 3).

**Table 3:** Microorganisms detected in this study.

**Tick species**	**Developmental stage/Gender**	**No. of specimens (No. of pools)**	**Host**	**Origin**	**Date of collection**	***Rickettsia***		***Coxiella***	***Rickettsiella***	***Francisella***	***Babesia***	***Adelina***
						**No. specimens (PI%)**	**No. of pools (MIR %)**	**No. of pools (MIR %)**	**No. of pools (MIR %)**	**No. of pools (MIR %)**	**No. of pools (MIR %)**	**No. of pools (MIR %)**
*Argas reflexus*	Nymphs	7 (2)	*Athene noctua*	Tabernas (Almería)	01/06/2018	0 (0)	0 (0)	**2 (28.6)**	0 (0)	0 (0)	0 (0)	0 (0)
	Nymph	1 (1)	*Coracias garrulus*	Tabernas (Almería)	28/06/2020	0 (0)	0 (0)	0 (0)	0 (0)	0 (0)	0 (0)	0 (0)
*Argas* sp. EEZA-CRETAV1	Nymph	1 (1)	*C. garrulus*	Tabernas (Almería)	31/05/2012	**1 (100)**	NP	0 (0)	0 (0)	**1 (100)**	0 (0)	0 (0)
	Males	2 (2)	*C. garrulus*	Uleila del Campo (Almería)	14/06/2015	**2 (100)**	NP	0 (0)	0 (0)	0 (0)	**1 (50)**	0 (0)
	Adults or nymphs (last stage)	3 (1)	*A. noctua*	Tabernas (Almería)	09/05/2018	**3 (100)**	NP	0 (0)	0 (0)	0 (0)	0 (0)	0 (0)
	Nymphs	6 (2)	*A. noctua*	Tabernas (Almería)	09/05/2018	**6 (100)**	NP	0 (0)	0 (0)	0 (0)	0 (0)	0 (0)
	Adult	1 (1)	*A. noctua*	Tabernas (Almería)	08/06/2018	**1 (100)**	NP	0 (0)	0 (0)	0 (0)	0 (0)	0 (0)
	Nymphs	3 (1)	*A. noctua*	Tabernas (Almería)	08/06/2018	**3 (100)**	NP	0 (0)	0 (0)	0 (0)	0 (0)	0 (0)
	Nymphs	12 (2)	*A. noctua*	Tabernas (Almería)	23/05/2019	**12 (100)**	NP	0 (0)	0 (0)	0 (0)	0 (0)	0 (0)
	Nymph	1 (1)^a^	*A. noctua*	Tabernas (Almería)	30/05/2019	**1 (100)**	NP	0 (0)	0 (0)	0 (0)	0 (0)	0 (0)
	Male	1 (1)^a^	*A. noctua*	Tabernas (Almería)	10/06/2019	**1 (100)**	NP	0 (0)	0 (0)	0 (0)	0 (0)	0 (0)
	Larva	1 (1)^a^	*A. noctua*	Tabernas (Almería)	17/06/2019	**1 (100)**	NP	0 (0)	0 (0)	0 (0)	0 (0)	0 (0)
	Nymphs	2 (1)^a^	*A. noctua*	Tabernas (Almería)	17/06/2019	**1 (50)**	NP	0 (0)	0 (0)	0 (0)	0 (0)	0 (0)
	Nymphs	5 (1)	*A. noctua*	Tabernas (Almería)	13/05/2020	**5 (100)**	NP	0 (0)	0 (0)	0 (0)	0 (0)	0 (0)
	Nymphs	7 (1)	*A. noctua*	Tabernas (Almería)	27/05/2020	**7 (100)**	NP	0 (0)	0 (0)	0 (0)	0 (0)	0 (0)
	Adult	1 (1)^d^	*A. noctua*	Tabernas (Almería)	11/06/2020	**1 (100)**	NP	0 (0)	0 (0)	0 (0)	0 (0)	0 (0)
	Nymphs	12 (2)^d^	*A. noctua*	Tabernas (Almería)	11/06/2020	**11 (91.6)**	NP	0 (0)	0 (0)	0 (0)	0 (0)	0 (0)
	Nymphs	25 (5)	*A. noctua*	Tabernas (Almería)	15/06/2020	**25 (100)**	NP	0 (0)	0 (0)	0 (0)	0 (0)	0 (0)
*Argas* sp. EEZA-CRETAV2	Male	1 (1)	*C. garrulus*	Tabernas (Almería)	2012	**1 (100)**	NP	0 (0)	0 (0)	0 (0)	0 (0)	0 (0)
	Nymph	1 (1)	*C. garrulus*	Uleila del Campo (Almería)	14/06/2015	**1 (100)**	NP	0 (0)	0 (0)	0 (0)	0 (0)	0 (0)
	Female	1 (1)	*C. garrulus*	Uleila del Campo (Almería)	14/06/2015	**1 (100)**	NP	**1 (100)**	0 (0)	0 (0)	0 (0)	0 (0)
	Nymphs	3 (1)	*A. noctua*	Tabernas (Almería)	09/05/2018	**3 (100)**	NP	0 (0)	0 (0)	0 (0)	0 (0)	0 (0)
	Nymphs	1 (1)	*C. garrulus*	Tahal (Almería)	18/06/2018	**1 (100)**	NP	0 (0)	**1 (100)**	0 (0)	0 (0)	0 (0)
	Nymphs	4 (1)	*A. noctua*	Tabernas (Almería)	23/05/2019	**4 (100)**	NP	0 (0)	0 (0)	0 (0)	0 (0)	0 (0)
	Nymphs	2 (1)^b^	*A. noctua*	Tabernas (Almería)	30/05/2019	**2 (100)**	NP	0 (0)	0 (0)	0 (0)	0 (0)	0 (0)
	Nymph	1 (1)^b^	*A. noctua*	Tabernas (Almería)	10/06/2019	**1 (100)**	NP	0 (0)	0 (0)	0 (0)	0 (0)	0 (0)
	Nymph	1 (1)^c^	*A. noctua*	Tabernas (Almería)	27/05/2020	**1 (100)**	NP	0 (0)	0 (0)	0 (0)	0 (0)	0 (0)
	Adult	1 (1)^c^	*A. noctua*	Tabernas (Almería)	11/06/2020	**1 (100)**	NP	0 (0)	0 (0)	0 (0)	0 (0)	0 (0)
	Nymphs	5 (1)^c^	*A. noctua*	Tabernas (Almería)	11/06/2020	**4 (80)**	NP	0 (0)	0 (0)	0 (0)	0 (0)	0 (0)
	Nymphs	12 (2)	*A. noctua*	Tabernas (Almería)	15/06/2020	**11 (91.6)**	NP	0 (0)	0 (0)	0 (0)	0 (0)	0 (0)
*Argas* sp. EEZA-CRETAV3	Nymphs	2 (1)	*C. garrulus*	Tabernas (Almería)	17/06/2009	0 (0)	0 (0)	**1 (50)**	0 (0)	0 (0)	0 (0)	0 (0)
	Nymph	1 (1)	*C. garrulus*	Tabernas (Almería)	08/06/2015	0 (0)	**1 (100)**	**1 (100)**	0 (0)	0 (0)	0 (0)	0 (0)
	Larvae	2 (1)	*C. garrulus*	Tabernas (Almería)	17/06/2018	0 (0)	0 (0)	**1 (50)**	0 (0)	0 (0)	0 (0)	0 (0)
	Larvae	7 (1)	*C. garrulus*	Tabernas (Almería)	27/06/2020	0 (0)	0 (0)	**1 (14.3)**	0 (0)	0 (0)	0 (0)	0 (0)
	Larvae	10 (2)	*C. garrulus*	Tabernas (Almería)	28/06/2020	0 (0)	**1 (10)**	**2 (20)**	0 (0)	0 (0)	0 (0)	**1 (10)**
*Argas* sp. EEZA-CRETAV4	larvae	2 (2)	*C. garrulus*	Pinarejos (Segovia)	15/07/2004	0 (0)	**2 (100)**	**1 (50)**	0 (0)	0 (0)	0 (0)	0 (0)
*Argas* sp. EEZA-CRETAV5	Males	2 (2)	*C. garrulus*	Illana (Guadalajara)	05/07/2004	0 (0)	**2 (100)**	0 (0)	0 (0)	0 (0)	0 (0)	0 (0)
Total						**112 (74.7)**	–	**10 (6.7)**	**1 (0.7)**	**1 (0.7)**	**1 (0.7)**	**1 (0.7)**
*A. reflexus*		8 (3)				0 (0)	0 (0)	**2 (25)**	0 (0)	0 (0)	0 (0)	0 (0)
*Argas* sp. EEZA-CRETAV1		83 (20)				**81 (97.6)**	NP	0 (0)	0 (0)	**1 (1.2)**	**1 (1.2)**	0 (0)
*Argas* sp. EEZA-CRETAV2		33 (10)				**31 (93.9)**	NP	1 (3)	**1 (3)**	0 (0)	0 (0)	0 (0)
*Argas* sp. EEZA-CRETAV3		22 (6)				0 (0)	**2 (9.1)**	**6 (27.3)**	0 (0)	0 (0)	0 (0)	**1 (4.5)**
*Argas* sp. EEZA-CRETAV4		2 (2)				0 (0)	**2 (100)**	**1 (50)**	0 (0)	0 (0)	0 (0)	0 (0)
*Argas* sp. EEZA-CRETAV5		2 (2)				0 (0)	**2 (100)**	0 (0)	0 (0)	0 (0)	0 (0)	0 (0)

PI, Prevalence of infection = (No. of positive ticks/total No. of individual ticks); MIR, Minimun infectious rate = (No. of positive pools/total No. of individual ticks) × 100; NP, not processed;

a,b,c,d: *same pool. Positive results are shown in bold*.

**Figure 2:**
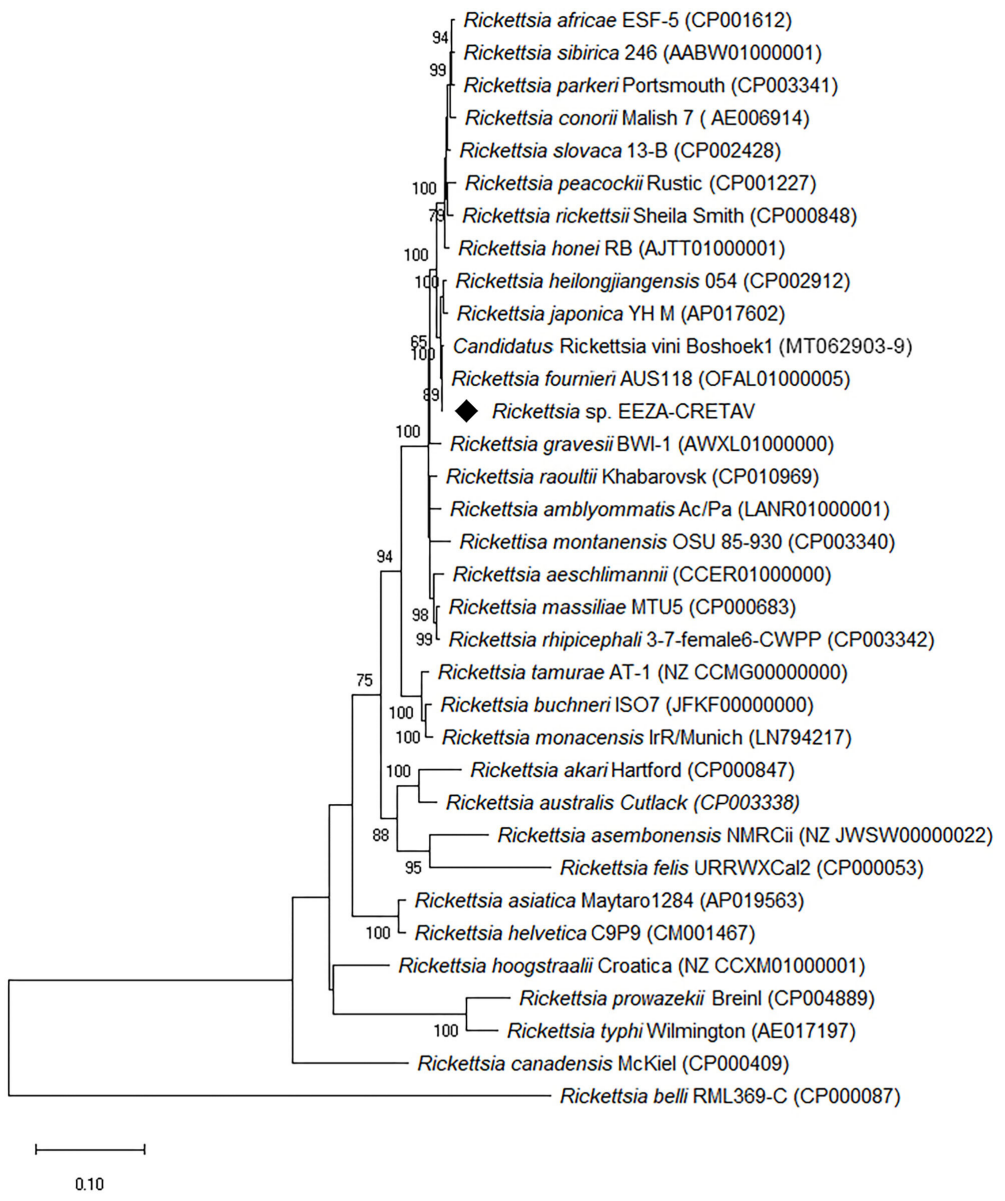
Phylogenetic tree showing the relationships between *Rickettsia* sp. EEZA-CRETAV and published *Rickettsia* spp. taxa. The evolutionary analysis was inferred using the maximum likelihood method and general time reversible + G model with Mega X, by concatenating fragments of six genes (*sca4*, 16s rRNA, *ompB, ompA*, 17-kDa, and *gltA*). The analysis involved 34 nucleotide sequences and a total of 4,120 positions in the final dataset. The tree is drawn to scale, with branch lengths measured in the number of substitutions per site. Numbers (>65%) shown at the nodes correspond to bootstrapped percentages (for 500 repetitions). The GenBank accession number of the sequences used in the analysis is shown in brackets after *Rickettsia* taxon name and the corresponding strain. Sequences obtained in this study are marked with diamond.

All the *ompA* gene sequences obtained were identical and showed the highest identity with *Rickettsia fournieri* (Table 4). Selected *Rickettsia*-positive samples were further genetically characterized by the amplification of five more rickettsial fragment genes (26). Nucleotide sequences for the respective genes were identical and showed highest identities with *R. fournieri* and *Candidatus* Rickettsia vini (Table 4). The phylogenetic tree based on the concatenated fragment genes of the *Rickettsia* strain detected, designated as *Rickettsia* sp. EEZA-CRETAV, corroborated the close relation with both *R. fournieri* and *Ca*. R. vini (Figure 2).

**Table 4:** Identities between fragment genes of *Rickettsia* sp. EEZA-CRETAV detected in the present study and published sequences from *Rickettsia fournieri* and *Candidatus* Rickettsia vini.

	**Identity (%)** **GenBank accession No**.				
	***ompA***	***ompB***	***gltA***	**16S rRNA**	**17-KDa**
*Rickettisia fournieri*	99.8 KF666477	99.7 KF666469	99.8 KF666473	99.7 KF666471	99.8 OFAL01000006
*Candidatus* Rickettsia vini	99.5 JF758828	99.6 MT062906	99.1 JF758829	99.8 JF803266	99.8 JF758827

A total of 27 nucleotide sequences were obtained using the *rpoB* PCR assay selected for the *Coxiella*/*Rickettsiella* detection but highest identities with validated bacterial species reached <85% for 14 samples. The genetic analysis of the amplicon obtained from an *Argas* sp. EEZA-CRETAV1 nymph, collected in Almería in 2012, showed the highest identity with *Francisella persicus* (PI = 0.7% and PI = 1.2% for *Argas* sp. EEZA-CRETAV1) (Tables 3, 5). The novel *Francisella* strain molecularly described in this study was designated as *Francisella* sp. EEZA-CRETAV. *Coxiella*-like strains were successfully amplified from 10 pools (MIR = 6.7%). Two out of three *A. reflexus* pools (with ticks collected in a little owl nest in Almería in 2018) showed infection with a *Coxiella* strain previously detected in this tick species (MIR = 25%) (Table 5). A new strain of *Coxiella* spp., designated as *Coxiella* sp. EEZA-CRETAV1, was amplified in all the pools of *Argas* sp. EEZA-CRETAV3 specimens (*n* = 6, MIR = 27.3%). One more *Coxiella* strain, *Coxiella* sp. EEZA-CRETAV2, was detected in two pools of different tick genotypes, an *Argas* sp. EEZA-CRETAV2 (MIR = 3%) female collected in Almería in 2015 and an *Argas* sp. EEZA-CRETAV4 (MIR = 50%) larva collected in Segovia in 2004 (Table 3). Nucleotide sequences corresponding to *rpoB* an *groEL* genes of these two novel strains shared 95.3 and 97% identity, respectively, and reached highest identities with *Coxiella* strains detected in *Ornithodoros* ticks (Table 5). Moreover, pools integrated over a single specimen were also submitted for *groEL* analysis and a nymph belonging to *Argas* sp. EEZA-CRETAV2, collected in Almería in 2018, showed infection with *Rickettsiella* sp. (PI = 0.7% and PI for *Argas* sp. EEZA-CRETAV2 = 3%; Table 3). The corresponding amplicon, designated as *Rickettsiella* EEZA-CRETAV, showed highest identities with *Rickettsiella* species amplified from *Ornithodoros normandi* (Table 5).

**Table 5:** Highest identities reached between fragment genes of *Francisella, Coxiella*, and *Rickettsiella* spp. detected in the present study and published sequences.

**Bacteria**	**Gene** **(GenBank accession No.)**	**Identity (%)** **Species** **(GenBank accession No.)**
*Francisella* sp. EEZA-CRETAV	*rpoB* MW287617	97.4 *Francisella persica* ATCC (CP013022, CP012505)
*Coxiella* sp. of *Argas reflexus*	*rpoB* MW287616	100 *Coxiella*-like endosymbiont of *Argas reflexus* (KY677983, KY677982)
*Coxiella* sp. EEZA-CRETAV1	*rpoB* MW287614	96.7 *Coxiella*-like endosymbiont of *Ornithodoros rostratus* (KP985288-91)
	*groEL* MW287611	97.0 Uncultured *Coxiella* sp. (KJ459055-6; detected in *Ornithodoros capensis*) *Coxiella*-like endosymbiont of *Ornithodoros peruvianus* (KP985466-7; KP985476-7)
*Coxiella* sp. EEZA-CRETAV2	*rpoB* MW287615	96.5 *Coxiella*-like endosymbiont of *Ornithodoros rostratus* (KP985288-91)
	*groEL* MW287612	97.4 *Coxiella*-like endosymbiont of *Ornithodoros amblus* (KP985447-8)
*Rickettsiella* sp. EEZA-CRETAV	*groEL* MW287613	99.7 *Rickettsiella* endosymbiont of *Ornithodoros normandi* (*KP985530*, KP985531)

All the tick pools were examined by PCR assays for the presence of Anaplasmataceae, *Bartonella*, and *Borrelia* species and all gave negative results.

### Viral Screening

Twenty-one pools originated from fresh/frozen ticks [*n* = 111 ticks: 1 tick (1 pool) of *A. reflexus*, 67 (12) of *Argas* sp. EEZA-CRETAV1, 26 (5) of *Argas* sp. EEZA-CRETAV2 and 17 (3) of *Argas* sp. EEZA-CRETAV3 specimens; Table 1] were analyzed for the presence of viruses belonging to families Flaviviridae, Orthonairoviridae, and Phenuiviridae. No sequences were amplified using the selected PCR assays.

### Protozoan Screening

**Figure 3:**
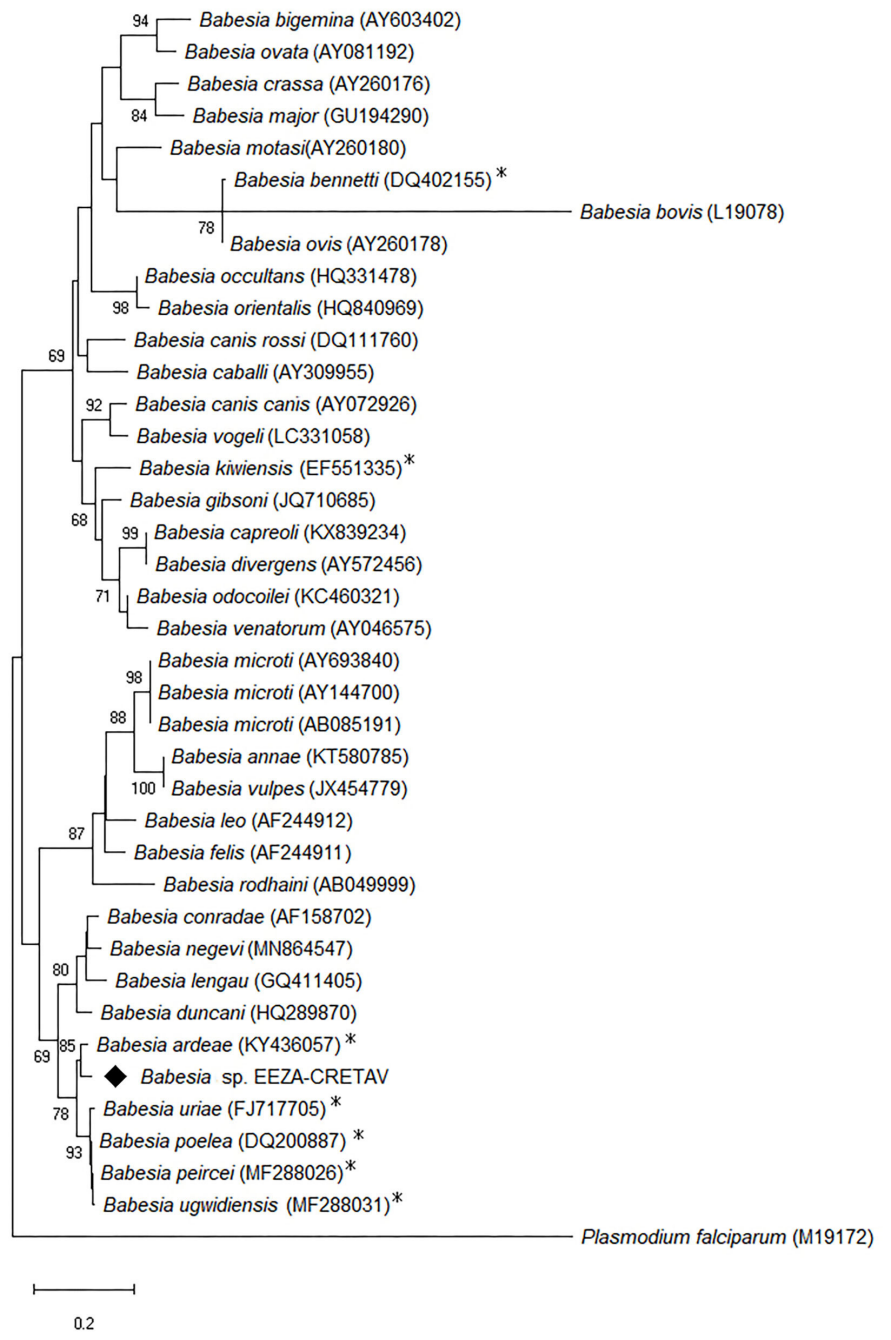
Maximum likelihood trees of *Babesia* species based on 18S rRNA analysis. The evolutionary analysis was inferred using Tamura-Nei model + G model with Mega X. The analysis involved 39 nucleotide sequences and a total of 483 positions in the final dataset. The tree is drawn to scale, with branch lengths measured in the number of substitutions per site. Numbers (>65%) shown at the nodes correspond to bootstrapped percentages (for 500 repetitions). The GenBank accession number of the sequences used in this analysis is shown in brackets after *Babesia* taxon name. The species found in this study is marked with diamond and the species detected in birds with asterisk. Taxon names are followed by GenBank accession numbers and collection location where available. *Plasmodium falciparium* is used as outgroup.

Tick DNA from 43 pools was analyzed using a PCR assay that amplifies 18S rRNA gene of *Babesia* and *Theileria* spp. (Table 3, Supplementary Table 1). *Babesia* sp. was detected from a male *Argas* sp. EEZA-CRETAV1 collected in Almería in 2015 (MIR = 0.7%, MIR = 1.2% for *Argas* sp. EEZA-CRETAV1) (Table 3). Three more ticks (one *Argas* sp. EEZA-CRETAV1 and two *Argas* sp. EEZA-CRETAV2 ticks) collected simultaneously from the same nest gave negative results. Based on the analysis of 18S rRNA gene, the closets *Babesia* sp. from the detected strain, designated as *Babesia* sp. EEZA-CRETAV, was *Babesia ardeae* (KY436057; 95.8% identity) (Figure 3). Two more genes, ITS1 and ITS2, were also examined, but currently there are no *B. ardeae* sequences available for these markers. The analysis of these genes showed highest (<82%) identities with *Babesia poelea* (accession no. DQ200887). A pool formed by 4 *Argas* sp. EEZA-CRETAV3 larvae, collected in Almería in 2020, showed presence of a coccidian parasite *Adelina bambarooniae* (AF494059) (MIR = 0.7%) (Table 3).

### Sequences Submission to a Public Database

The nucleotide sequences of ticks and microorganisms detected in this study (*n* = 42) were deposited in the GenBank database under accession numbers showed in Table 6.

**Table 6:** GenBank accession numbers of the nucleotide sequences obtained in this study, differing from published sequences.

**Organisms**	**Target gene**	**Accession no**
*Argas reflexus*	16S rRNA	MW289075; MW289076
	12S rRNA	MW289084
	COI	MW288388; MW288389
*Argas* sp. EEZA-CRETAV1	16S rRNA	MW289069
	12S rRNA	MW289077
	COI	MW288380; MW288381
*Argas* sp. EEZA-CRETAV2	16S rRNA	MW289070; MW289071
	12S rRNA	MW289078; MW289079; MW289080
	COI	MW288382; MW288383; MW288384
*Argas* sp. EEZA-CRETAV3	16S rRNA	MW289072
	12S rRNA	MW289081; MW289082
	COI	MW288385
*Argas* sp. EEZA-CRETAV4	16S rRNA	MW289073
	12S rRNA	MW289083
	COI	MW288386
*Argas* sp. EEZA-CRETAV5	16S rRNA	MW289074
	COI	MW288387
*Ricekttsia* sp. EEZA-CRETAV	*ompA*	MW287618
	*ompB*	MW287619
	*sca4*	MW287620
	*gltA*	MW287621
	16S rRNA	MW296096
	17-kDa	MW287622
*Coxiella* sp. EEZA-CRETAV1	*rpoB*	MW287614
	*groEL*	MW287611
*Coxiella* sp. EEZA-CRETAV2	*rpoB*	MW287615
	*groEL*	MW287612
*Coxiella* sp. from *Argas reflexus*	*rpoB*	MW287616
*Francisella* sp. EEZA-CRETAV	*rpoB*	MW287617
*Rickettsiella* sp. EEZA-CRETAV	*groEL*	MW287613
*Babesia* sp. EEZA-CRETAV	18S rRNA	MW287597
	ITS1	MW287607
	ITS2	MW287606

## Discussion

Soft ticks are important vectors of microbial agents of animal and human diseases. Despite this, the knowledge of argasid tick species and their associated microorganisms is generally scarce. Herein, the occurrence of five novel *Argas* spp. genotypes, in addition to *A. reflexus*, collected in the Iberian Peninsula (Spain) in nests occupied by little owls and European rollers is reported. Moreover, we detected the presence of tick-borne microorganisms belonging to genera *Rickettsia, Coxiella, Francisella, Rickettsiella*, and *Babesia* in *Argas* ticks. In contrast, Anaplasmataceae, *Bartonella*, and *Borrelia* bacterium species and viruses belonging to the families Flaviviridae, Orthonairoviridae, and Phenuiviridae have not been detected.

### Tick Identification

Of the six *Argas* genotypes detected in this study, only one could be identified to the species level, namely, *A. reflexus*. The morphological identification of ticks is challenging, even for experts (27), while the molecular approach appears to be an accurate tool for tick identification (25, 27). Nevertheless, increased effort for molecular characterization of more *Argas* species is needed for a reliable taxonomic inference based on molecular tools. In order to confirm if the genotypes identified in this study represent validated or potentially novel tick species, a further morphological analysis including unfed larva specimens, should be performed along with rigorous molecular analyses of *Argas* ticks from diverse geographical locations.

*Argas reflexus* specimens, known as the pigeon tick, have been collected in two bird nests in Almería (Southern Spain), one occupied by little owl (*n* = 7) and one by roller (*n* = 1). This species occurs in Spain, and it is a well-known ectoparasite of little owls, whereas information regarding roller infestations is scarce (6, 20, 28). *Argas* sp. EEZA-CRETAV4 and *Argas* sp. EEZA-CRETAV5, genotypes amplified in Central Spain, clustered molecularly with *A. persicus* from China (29), but in a different branch than other *A. persicus* specimens (Figure 1). Nevertheless, the broad genetic divergence of this group (29, 30), also revealed in the phylogeny inferred herein (Figure 1), suggests that cryptic species could occur in this taxon known as fowl tick. This worldwide distributed tick has been previously reported in Spain and is known to infest wild birds also in other countries (6, 28). In addition, ticks of three more *Argas* genotypes have been identified, all of them in Almería. The *Argas* sp. EEZA-CRETAV1–2 genotypes clustered together and appear to be closely related with *A. vulgaris* and *A. polonicus*, respectively (Figure 1). Neither of these two tick species have been previously reported from western Europe and their occurrence is only documented in a few eastern European countries (20, 21, 31). These genotypes have been found in the nest material of roller and little owls, occurring in the same nests in several cases (Table 1). In the study area, there is a high competition among cavity-nesting birds for suitable cavities and the same cavity can be successively used by little owls, pigeons and rollers (32). This fact could explain the infestation of both bird species by the same nidicolous tick taxa, i.e., *A. reflexus* and *Argas* sp. EEZA-CRETAV1–2. In contrast, *Argas* sp. EEZA-CRETAV3, closely related to *A. africolumbae*, has been found only in nests occupied by rollers. The roller is a long-distant migrant species (trans-Saharan migrant) and the Spanish populations overwinter in different African regions (33). It is worth mentioning that *A. africolumbae* is an ornithophilic tick that occurs in Africa: South Africa, Kenya, Tanzania and Burkina Faso (34, 35). It is well-known that birds can serve as dispersers of ticks and tick-borne microorganisms, even though this information pertains mainly to hard ticks (36, 37). Some studies suggest that the role of birds as dispersers of soft ticks is less important, due to the biology of these ticks (nidicolous behavior and shorter blood-feeding time), but the role of migratory birds as reservoirs or amplifiers of tick-borne microorganisms associated with soft ticks remains to be better investigated (38, 39).

### Tick-Borne Microorganisms

The microbiological screening of ticks is important to identify the local risks of emergence of tick-borne diseases. *Argas* species, including *A. reflexus* and *A. persicus*, have been described as important pests and vectors of diseases in poultry and wild birds, being responsible for high economic losses (7, 11, 40). These tick species have also been recorded biting humans and causing anaphylaxis episodes (8, 10). Although humans are accidental hosts of *Argas* ticks and the ticks carry numerous microorganisms, human pathogens among them, the role of these ticks as vectors of human infectious agents has not been proven.

The most prevalent microorganisms amplified in this study belong to the *Rickettsia* genus (α-Proteobacteria; Rickettsiaceae). The high prevalence of *Rickettsia* spp. found in ticks of *Argas* sp. EEZA-CRETAV1–2 specimens suggests that the bacterium is a common endosymbiont of ticks of the two genotypes. The detection of the newly described *Rickettsia* strain in tick-body samples, but not in tick-leg samples of ticks of other genotypes (*Argas* sp. EEZA-CRETAV3–5), suggests that this *Rickettsia* species may not be a true intracellular endosymbiont and its presence in the former genotypes could be acquired through feeding on infected hosts or by cofeeding. The phylogenetic analysis of *Rickettsia* sp. EEZA-CRETAV reveals its close relation with *R. fournieri* and *Ca*. R. vini, both *Rickettsia* species associated with ornithophilic nidicolous ticks (41, 42). While *R. fournieri* has been described only once from *A. lagenoplastis* in Australia (41), *Ca*. R. vini has been detected in several European countries in *Ixodes* spp. (36, 42–45). The single study performed suggests that *Ca*. R. vini is not pathogenic (43). Nevertheless, the two *Rickettsia* taxa are closely related to other *Rickettsia* species that are recognized as human pathogens, specifically *Rickettsia japonica* and *Rickettsia heilongjiangensis* (46, 47). Thus, the epidemiology and pathological potential of *Rickettsia* strains such as *Rickettsia* sp. EEZA-CRETAV, in addition to *R. fournieri* and *Ca*. R. vini, should be further investigated. Likewise, the isolation of *Rickettsia* sp. EEZA-CRETAV is necessary to gain an insight into the epidemiological importance of this strain.

In addition to the *Rickettsia* taxon, this study has revealed for the first time different proteobacterial tick endosymbionts in *Argas* spp. from Spain, namely, *Coxiella, Rickettsiella* (Gamma-proteobacterium; Coxiellaceae) and *Francisella* (Gamma-proteobacterium; Francisellaceae) species. The detected species, commonly known as *Rickettsiella*-like, *Coxiella*-like, and *Francisella*-like, are intracellular obligatory endosymbionts important for tick survival. They play some role in B vitamins biosynthesis and their presence may interfere with the transmission (positively or negatively) of other microorganisms, including tick-borne pathogens (48). They are related to species responsible for important human diseases. For instance, *Coxiella burnetii* and *Francisella tularensis* cause Q fever and tularemia, respectively (49, 50). *Coxiella*-like species have been implicated in human and avian diseases (51, 52). Three different *Coxiella* strains have been successfully detected in this study. Of them, the *Coxiella* sp. from *A. reflexus* was homologous to the species previously detected in the same tick species, but the remaining two strains, designated as *Coxiella* sp. EEZA-CRETAV1–2, differ from the scarcely-known *Coxiella*-like strains of *Argas* species (14, 53–55). To date, *Rickettsiella* has been mainly reported in hard ticks (*Ixodes* spp. and *Haemaphysalis* spp.) and soft ticks belonging to the genus *Ornithodoros* (14). The presence of *Rickettsiella* in *Argas* ticks has been suggested for the bat tick *A. transgariepinus* from Namibia, but the available 16S rRNA sequences shared low identities with the published *Rickettsiella* sequences (17). In the present study, a *Rickettsiella* sp. similar to that of *O. normandi* from Tunisia (55) was detected in an *Argas* sp. EEZA-CRETAV2 specimen. Lastly, a strain closely related to *F. persicus* was found in an *Argas* sp. EEZA-CRETAV1 specimen. *Francisella persicus*, formerly *Wolbachia persica*, is an endosymbiont of *Argas arboreus* (previously referred to as *A. persicus*) (56). This bacterium has not been identified as an animal or human pathogen, but the analysis of its genome shows that this species conserves an important number of potentially functional virulence-associated genes, suggesting that it could be pathogenic to mammals (57).

Bacteria of Anaplasmataceae family, *Bartonella*, and *Borrelia* spp. have not been detected in the ticks analyzed. *Argas* spp. are recognized vectors of *B. anserine*, the agent of the avian spirochetosis, a worldwide distributed disease of veterinary importance that has not been reported from Spain (11, 40). The lack of virus detection in this study was unexpected, because diverse viruses are readily detected in *Argas* ticks (7). Scarce tick-borne viruses have been described from Spain and all but Meaban-like virus, a flavivirus found in *Ornithodoros maritimus* (38), are associated to ixodid ticks. Some of these viruses have a great relevance for human health, e.g., Crimean-Congo hemorrhagic fever virus, whose arrival in infected ticks has been suggested to take place through migratory birds (37, 58). This fact highlights the importance of studying viruses in soft ticks associated to birds in Spain, an important area in the migratory routes of many avian species, because what it is not sought, it is not found (59).

Two apicomplexan parasites have been found in this work, *Babesia* sp. and *A*. *bambarooniae*, though the latter species is not known as a tick-borne microorganism. In turn, the apicomplexan piroplasms *Babesia* (Aconoidasida; Babesiidae) are mainly vectored by ixodid ticks, though argasid ticks also were suggested as potential vectors (60). *Babesia* sp. EEZA-CRETAV has been amplified from an *Argas* sp. EEZA-CRETAV3 tick associated with rollers and the presence of the protozoan in the blood of rollers cannot be rejected. Sixteen *Babesia* species responsible for avian piroplasmosis, in addition to several strains that are not fully identified, are known (61). Of these, *Babesia frugilegica, Babesia shortti* and *Babesia benneti* have been reported from Spain (62–64). *Babesia* sp. EEZA-CRETAV is closely related to some of the scarcely genetically characterized *Babesia* species, mainly to *B. ardeae* (Figure 3). This species has been detected in Asia and its pathogenicity is unknown (61). This strain is also close to the human-pathogenic *B. ducani* that has been identified in North America, the United Kingdom and Australia (65). To our knowledge, *Babesia* spp. have not been identified in owls.

## Conclusions

The sedentary lifestyle of soft ticks could imply a limited role of these ticks in the circulation of infectious agents (66). However, as indicated by this study, the high re-use of cavities within and between years by different bird species could importantly enhance the spread of microorganisms associated with soft nidicolous ticks, such as *Argas* ticks.

This study highlights the richness of nidicolous *Argas* ticks associated with cavity-nesting birds in a semi-desert area in Western Europe, and suggests that the diversity of this genus in Spain might be underestimated. Moreover, this work provides the first report of *Rickettsia* sp., *Coxiella* spp., *Rickettsiella* sp., *Francisella* sp. and *Babesia* sp., from soft ticks in Spain, and *A. bambarooniae* from Ixodida.

Further research should be carried out to confirm if the new genotypes of ticks and their microorganisms represent novel taxa and, additionally, to understand their role in the epidemiology of zoonoses using the One Health approach.

## Data Availability Statement

The datasets presented in this study can be found in online repositories. The names of the repository/repositories and accession number(s) can be found in the article/Supplementary Material.

## Author Contributions

APa, RV, JO, and FV designed the initial study. JV and FV carried out the field work. APa performed the tick identification and tick processing. APa, APo, SS, and PS implemented the analysis of microorganisms. APa and FV wrote the first draft of the manuscript. All authors contributed to data interpretation and revisions.

## Conflict of Interest

The authors declare that the research was conducted in the absence of any commercial or financial relationships that could be construed as a potential conflict of interest.
