# Supplementary material for: Novel Genotypes of Nidicolous Argas Ticks and Their Associated Microorganisms From Spain
Source: Front Vet Sci. 2021 Mar 29;8:637837. doi: 10.3389/fvets.2021.637837 (PMC8039128; doi:10.3389/fvets.2021.637837)
Supplement: Supplementary file 1 [file Table_1.docx]

**Novel genotypes of nidicolous *Argas* ticks and their associated microorganisms from Spain.**

**Supplementary table.** PCR primer pairs and conditions used in this study.

| **Organisms** | **Target gene** | **Primer sequence (5’🡪 3’)** | **Fragment**  **size (bp)** | **Tm (ºC)** | **Reference** |
| --- | --- | --- | --- | --- | --- |
| **Ticks** | 16S rRNA | F:CTGCTCAATGATTTTTTAAATTGCTGTGG  R:CCGGTCTGAACTCAGATCAAGT | 456 | 48  54 | Black and Piesman, 1994 |
|  | 12S rRNA^1^ | F:AAACTAGGATTAGATACCCT  R:AATGAGAGCGACGGGCGATGT | 338 | 51  53 | Beati and Keirans, 2001 |
|  | COI^1^ | F:GGTCAACAAATCATAAAGATATTGG  R:TAAACTTCAGGGTGACCAAAAAATCA | 710 | 40 | Folmer et al., 1994 |
| ***Anaplasma/Ehrlichia*/ *Neoehrlichia* spp.** | *gro*ESL | F:AITGGGCTGGTAITGAAAT  R:CCICCIGGIACIAIACCTTC | 1,350 | 48 | Liz et al., 2002 |
|  |  | F:ATWGCWAARGAAGCATAGTC  R:CTCAACAGCAGCTCTAGTAGC | 1297 | 75 |  |
| ***Bartonella* spp.** | *rpoB* | F:CGCATTGGCTTACTTCGTATG  R:GTAGACTGATTAGAACGCTG | 825 | 53 | Renesto et al., 2001 |
| ***Borrelia* spp.** | *glpQ* (Relapsing fever group) | F:ATGGGTTCAAACAAAAAGTCACC  R:CATTACTGTGTCAGTAAAATCTGTAAATATACCATCTAC | 920 | 70 Touchdown  60 | Hovious et al., 2013 |
|  |  | F:ATGGGTTCAAACAAAAAGTCACC  R:CCAGGGTCCAATTCCATCAGAATATTGTGCAAC | 700 | 53 | Wagemakers et al., 2017 |
|  | 16S rRNA | F: GCTGGCAGTGCGTCTTAAGC  R: GCTTCGGGTATCCTCAACTC | 1350 | 55 | Raoult et al., 1998 |
|  | *flaB* | F: TAATACGTCAGCCATAAATGC  R: GCTCTTTGATCAGTTATCATTC | 750 | 56 | Assous et al., 2006 |
| ***Coxiella* /*Rickettsiella* spp.** | *rpoB* | F:GGGCGNCAYGGWAAYAAAGGSGT  R:CACCRAAHCGTTGACCRCCAAATTG | 619 | 56 | Duron et al., 2015 |
|  |  | F:TCGAAGAYATGCCYTATTTAGAAG  R:AGCTTTMCCACCSARGGGTTGCTG | 542 | 56 |  |
|  | *groEL*^1^ | F:TTTGAAAAYATGGGCGCKCAAATGGT  R:CGRTCRCCAAARCCAGGTGC | 655 | 56 | Duron et al., 2015 |
|  |  | F:GAAGTGGCTTCGCRTACWTCAGACG  R:CCAAARCCAGGTGCTTTYAC | 619 | 56 |  |
| **Spotted Fever group *Rickettsia* spp.** | *ompA* | F:ATGGCGAATATTTCTCCAAAA  R:GTTCCGTTAATGGCAGCATCT | 631 | 46 | Roux et al., 1996  Regnery et al., 1991 |
|  |  | F:ATGGCGAATATTTCTCCAAAA  R:AGTGCAGCATTCGCTCCCCCT | 532 | 48 |  |
|  | *ompB*^1^ | F:AAACAATAATCAAGGTACTGT  R:TACTTCCGGTTACAGCAAAGT | 811 | 55 | Roux and Raoult, 2000 |
|  | *sca4*^1^ | F:ATGAGTAAAGACGGTAACCT  R:AAGCTATTGCGTCATCTCCG | 928 | 50 | Sekeyova et al., 2001 |
|  | *gltA* ^1^ | F: GGGGGCCTGCTCACGGCGG  R:ATTGCAAAAAGTACAGTGAACA | 1019 | 65 | Jado et al., 2007 |
|  | gltA (5’ end) ^1^ | F: GCAAGTATCGGTGAGGATGTAAT R:GCTTCCTTAAAATTCAATAAATCAGGAT | 401 | 48 | Labruna et al., 2004 |
|  | 16S rRNA^1^ | F:AGAGTTTGATCCTGGCTCAG  R:AACGTCATTATCTTCCTTGC | 426 | 59 | Weisburg et al., 1991 ; Marquez, 1998 |
|  | 17-kDa | F:GCTCTTGCAACTTCTATGTT  R:CATTGTTCGTCAGGTTGGCA | 434 | 58 | Oliveira et at., 2002 |
| **Flaviviridae**^2^ | NS5 | F:TGYRTBTAYAACATGATGG  R:GTGTCCCAICCNGCNGTRTC | 269-272 | 50 | Moureau et al., 2007 |
| NS5 | F:GTGTCCCAGCCGGCGGTGTCATCAGC  R:AACATGATGGGRAARAGRGARAA | 252 | 53 | Scaramozzino et al., 2001 |  |
|  | F:GTGTCCCAGCCGGCGGTGTCATCAGC  R:AARGGHAGYMCDGCHATHTGGT | 220 | 54 |  |  |
| **Orthonairoviridae^2^** | S Segment | F:TCTCAAAGAAACACGTGCCGC  R:GTCCTTCCTCCACTTGW | 400 | 55 | Lambert and Lanciotti, 2009 |
| **Phenuiviridae**^2^ | L Segment | F:CAGCATGGIGGICTIAGAGAGAT  R:TGIAGIATSCCYTGCATCAT | 514 | 55 | Matsuno et al., 2015 |
|  | L Segment | F:GGCTACTTCAARAAYAARGANGA  R:CTCTCTCAGICCICCRTGYTG | 507 | 50 | Klimentov et al., 2016 |
| ***Babesia*/ *Theileria* spp.** | 18S rRNA | F:GACACAGGGAGGTAGTGACAAG  R:CTAAGAATTTCACCTCTGACAGT | 400 | 51 | Gubbels et al., 1999 |
|  | ITS1^1^ | F:CGAGTGATCCGGTGAATTATTC  R:CCTTCATCGTTGTGTGAGCC | 615 | 52 | Blaschitz et al., 2008; Bajer et al., 2014 |
|  | ITS2^1^ | F:GGCTCACACAACGATGAAGG  R:CTCGCCGTTACTAAGGGAATC | 315 | 54 | Blaschitz et al., 2008; Bajer et al, 2014 |

F: Forward; R: Reverse; bp: base pairs; Tm: melting temperature; N= A/C/G/T; Y= C/T; K: G/T; W:A/T; R: G/A; B: C/G/T; S: G/C; H: A/C/T;

^1^:Performed only with selected samples that gave positive results in the analysis of the corresponding microorganism group using the screening gene.

^2^:Performed only with pools formed by samples that were preserved frozen

# References

Assous, M.V., Wilamowski, A., Bercovier, H., Marva, E. (2006). Molecular characterization of tickborne relapsing fever *Borrelia*, Israel.

Emerg Infect Dis. 12:1740-1743. doi: 10.3201/eid1211.060715.

Bajer, A., Alsarraf, M., Bednarska, M., Mohallal, E.M., Mierzejewska, E.J., Behnke-Borowczyk, J., Zalat, S., Gilbert, F., Welc-Falęciak, R. (2014). *Babesia behnkei* sp. nov., a novel *Babesia* species infecting isolated populations of Wagner's gerbil, *Dipodillus dasyurus*, from the Sinai Mountains, Egypt. Parasit Vectors. 7:572. doi: 10.1186/s13071-014-0572-9.

Black, W.C., and Piesman, J. (1994). Phylogeny of hard and soft tick taxa (Acari:Ixodida) based on mitochondrial 16S rDNA sequences. Proc. Natl. Acad. Sci. USA. 91:10034-100348.

Beati, L., and Keirans, J.E. (2001). Analysis of the systematic relationships among ticks of the genera *Rhipicephalus* and *Boophilus* (Acari: Ixodidae) based on mitochondrial 12S ribosomal DNA gene sequences and morphological characters. J Parasitol. 87:32-48.

Blaschitz, M., Narodoslavsky-Gföller, M., Kanzler, M., Stanek, G., Walochnik, J. (2008). *Babesia* species occurring in Austrian *Ixodes ricinus* ticks. Appl Environ Microbiol. 74(15):4841-4846. doi: 10.1128/AEM.00035-08.

Duron, O., Noël, V., McCoy, K.D., Bonazzi, M., Sidi-Boumedine, K., Morel, O., Vavre, F., Zenner, L., Jourdain, E., Durand, P., Arnathau, C., Renaud, F., Trape, J.F., Biguezoton, A.S., Cremaschi, J., Dietrich, M., Léger, E., Appelgren, A., Dupraz, M., Gómez-Díaz, E., Diatta, G., Dayo, G.K., Adakal, H., Zoungrana, S., Vial, L., Chevillon, C. (2015). The Recent Evolution of a Maternally-Inherited Endosymbiont of Ticks Led to the Emergence of the Q Fever Pathogen, *Coxiella burnetii*. PLoS Pathog. 11(5):e1004892. doi: 10.1371/journal.ppat.1004892.

Folmer, O., Black, M., Hoeh, W., Lutz, R., Vrijenhoek, R. (1994). DNA primers for amplification of mitochondrial cytochrome c oxidase subunit I from diverse metazoan invertebrates. Mol Mar Biol Biotechnol. 3(5):294-299.

Gubbels, J.M., de Vos, A.P., van der Weide, M., Viseras, J., Schouls, L.M., de Vries, E., Jongejan, F. (1999). Simultaneous detection of bovine *Theileria* and *Babesia* species by reverse line blot hybridization. J Clin Microbiol. 37(6):1782-1789.

Hovius, J.W., de Wever, B., Sohne, M., Brouwer, M.C., Coumou, J., Wagemakers, A., Oei, A., Knol, H., Narasimhan, S., Hodiamont, C.J., Jahfari, S., Pals, S.T., Horlings, H.M., Fikrig, E., Sprong, H., van Oers, M.H. (2013). A case of meningoencephalitis by the relapsing fever spirochaete *Borrelia miyamotoi* in Europe. Lancet. 382:658.

Jado, I., Oteo, J.A., Aldámiz, M., Gil, H., Escudero, R., Ibarra, V., Portu, J., Portillo, A., Lezaun, M.J., García-Amil, C., Rodríguez-Moreno, I., Anda, P. (2007). *Rickettsia monacensis* and human disease, Spain. Emerg Infect Dis. 13:1405-1407.

Klimentov A.S., Butenko A.M., Khutoretskaya N.V., Shustova E.Y., Larichev V.F., Isaeva O.V., Karganova G.G., Lukashev A.N., Gmyl A.P. (2016). Development of pan-phlebovirus RT-PCR assay. J Virol Methods. 232:29-32. doi: 10.1016/j.jviromet.2016.02.009.

Labruna, M.B., Whitworth, T., Horta, M.C., Bouyer, D.H., McBride, J.W., Pinter, A., Popov, V., Gennari, S.M., Walker, D.H. (2004). *Rickettsia* species infecting *Amblyomma cooperi* ticks from an area in the state of São Paulo, Brazil, where Brazilian spotted fever is endemic. J Clin Microbiol. 42:90-98.

Lambert, A.J., and Lanciotti R.S. (2009). Consensus amplification and novel multiplex sequencing method for S segment species identification of 47 viruses of the Orthobunyavirus, Phlebovirus, and Nairovirus genera of the family Bunyaviridae. J Clin Microbiol. 47(8):2398-2404. doi: 10.1128/JCM.00182-09.

Liz, J.S., Sumner, J.W., Pfister, K., Brossard, M. (2002). PCR detection and serological evidence of granulocytic ehrlichial infection in roe deer (*Capreolus capreolus)* and chamois (*Rupicapra rupicapra*). J Clin Microbiol. 40:892–897. doi: 10.1128/JCM.40.3.892-897.2002

Márquez, F.J., Muniain, M.A., Soriguer, R.C., Izquierdo, G., Rodríguez-Baño, J., Borobio, M.V. (1998). Genotypic identification of an undescribed spotted fever group rickettsia in *Ixodes ricinus* from southwestern Spain. Am J Trop Med Hyg. 58: 570-577.

Matsuno, K., Weisend, C., Kajihara, M., Matysiak, C., Williamson, B.N., Simuunza, M., Mweene, A.S., Takada, A., Tesh, R.B., Ebihara, H. (2015). Comprehensive molecular detection of tick-borne phleboviruses leads to the retrospective identification of taxonomically unassigned bunyaviruses and the discovery of a novel member of the genus phlebovirus. J Virol. 89(1):594-604. doi: 10.1128/JVI.02704-14.

Moureau, G., Temmam, S., Gonzalez, J.P., Charrel, R.N., Grard, G., de Lamballerie, X. (2007). A real-time RT-PCR method for the universal detection and identification of flaviviruses. Vector Borne Zoonotic Dis. 7(4):467-477. doi: 10.1089/vbz.2007.0206.

Oliveira, R.P., Galvão, M.A., Mafra, C.L., Chamone, C.B., Calic, S.B., Silva, S.U., Walker, D.H. (2002). *Rickettsia felis* in *Ctenocephalides* spp. fleas, Brazil. Emerg Infect Dis. 8:317-319.

Raoult, D., Ndihokubwayo, J.B., Tissot-Dupont, H., Roux, V., Faugere, B., Abegbinni, R., Birtles, R.J. (1998). Outbreak of epidemic typhus associated with trench fever in Burundi. Lancet. 352(9125):353-358. doi: 10.1016/s0140-6736(97)12433-3.

Regnery, R.L., Spruill, C.L., Plikaytis, B.D. (1991). Genotypic identification of rickettsiae and estimation of intraspecies sequence divergence for portions of two rickettsial genes. J Bacteriol. 173:1576-1589.

Renesto, P., Gouvernet, J., Drancourt, M., Roux, V., Raoult, D. (2001). Use of rpoB gene analysis for detection and identification of Bartonella species. J Clin Microbiol. 39:430-437.

Roux, V., Fournier, P.E., Raoult, D. (1996). Diffentiation of Spotted Fever Group Rickettsiae by Sequencing and Analysis of Restriction Fragment Length Polymorphism of PCR-Amplified DNA of the Gene Encoding the Protein rOmpA. J Clin Microbiol. 34:2058-2065.

Roux, V., and Raoult, D. (2000). Phylogenetic analysis of members of the genus *Rickettsia* using the gene encoding the outer-membrane protein rOmpB (*ompB*). Int J Syst Evol Microbiol. 50 Pt 4: 1449-1455.

Scaramozzino, N., Crance, J.M., Jouan, A., DeBriel, D.A., Stoll, F., Garin, D. (2001). Comparison of flavivirus universal primer pairs and development of a rapid, highly sensitive heminested reverse transcription-PCR assay for detection of flaviviruses targeted to a conserved region of the NS5 gene sequences. J Clin Microbiol. 39(5):1922-1927. doi: 10.1128/JCM.39.5.1922-1927.2001.

Sekeyova, Z., Roux, V., Raoult, D. (2001). Phylogeny of *Rickettsia* spp. Inferred by comparing sequences of ‘gene D’, which encodes an intracytoplasmic protein. Int. J. Syst. Evol. Microbiol. 51:1353-1360.

Wagemakers, A., Jahfari, S., de Wever, B., Spanjaard, L., Starink, M.V., de Vries, H.J., Sprong, H., Hovius, J.W. (2017). *Borrelia miyamotoi* in vectors and hosts in The Netherlands. Ticks Tick Borne Dis. 8(3):370-374.

Weisburg, W.G., Barns, S.M., Pelletier, D.A., Lane, D.J. (1991). 16S ribosomal DNA amplification for phylogenetic study. J Bacteriol. 173:697-703.
